# Efficacy of the FDA nozzle benchmark and the lattice Boltzmann method for the analysis of biomedical flows in transitional regime

**DOI:** 10.1007/s11517-020-02188-8

**Published:** 2020-06-07

**Authors:** Kartik Jain

**Affiliations:** Faculty of Engineering Technology, University of Twente, P.O. Box 217, 7500AE Enschede, The Netherlands

**Keywords:** Lattice Boltzmann method, Transitional flow, Turbulence, FDA, Nozzle, Hydrodynamic instability

## Abstract

Flows through medical devices as well as in anatomical vessels despite being at moderate Reynolds number may exhibit transitional or even turbulent character. In order to validate numerical methods and codes used for biomedical flow computations, the US Food and Drug Administration (FDA) established an experimental benchmark, which was a pipe with gradual contraction and sudden expansion representing a nozzle. The experimental results for various Reynolds numbers ranging from 500 to 6500 were publicly released. Previous and recent computational investigations of flow in the FDA nozzle found limitations in various CFD approaches and some even questioned the adequacy of the benchmark itself. This communication reports the results of a lattice Boltzmann method (LBM) – based direct numerical simulation (DNS) approach applied to the FDA nozzle benchmark for transitional cases of Reynolds numbers 2000 and 3500. The goal is to evaluate if a simple *off the shelf* LBM would predict the experimental results without the use of complex models or synthetic turbulence at the inflow. LBM computations with various spatial and temporal resolutions are performed—in the extremities of 45 million to 2.88 billion lattice cells—executed respectively on 32 CPU cores of a desktop to more than 300,000 cores of a modern supercomputer to explore and characterize miniscule flow details and quantify Kolmogorov scales. The LBM simulations transition to turbulence at a Reynolds number 2000 like the FDA’s experiments and acceptable agreement in *jet breakdown locations, average velocity, shear stress*, and *pressure* is found for both the Reynolds numbers.

**Electronic supplementary material:**

The online version of this article (10.1007/s11517-020-02188-8) contains supplementary material, which is available to authorized users.

**Graphical Abstract:**
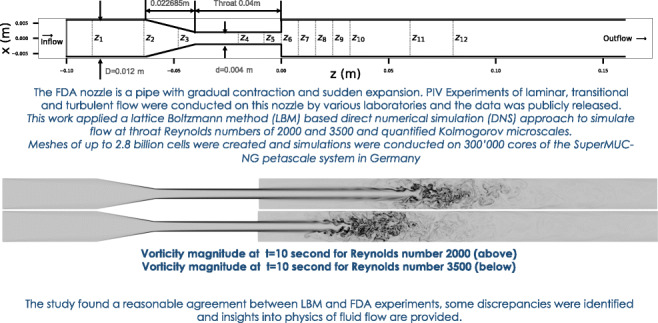
A bisecting plane showing the FDA nozzle and vorticity magnitude at t = 10 s for throat Reynolds numbers of 2000 and 3500

## Introduction

Computational fluid dynamics (CFD) has seen a growing interest in the biomedical community as flows within the cardiovascular system or in medical devices can be computed with sufficient ease to analyze various clinically and physiologically relevant details. However, the results from CFD can only be relied if the methods and software tools have been comprehensively verified and validated. The presence of turbulence in devices as well as in physiological flows pose additional challenges for the validation of CFD tools due to the complexity of the transitional flow physics itself. Furthermore, an appropriate quantification of such a flow regime requires fully resolved direct numerical simulation (DNS).

**Table 1:** Computational studies of flow in the FDA nozzle geometry in reverse chronological order

Author group	*Re*_*th*_ 500	*Re*_*th*_ 2000	*Re*_*th*_ 3500	*Re*_*th*_ 5000	*Re*_*th*_ 6500	Numerical method	Turbulence model	*Re*_*crit*_
Present work	Θ	✓	✓	Θ	Θ	LBM DNS	None	2000
Fehn et al. [7]	✓	✓	✓	✓	✓	High-order DG	None	$\sim $ 2400
Bergersen et al. [1]	Θ	Θ	✓	Θ	Θ	Low-order FEM	None	3500
Nicoud et al. [19]	✓	✓	✓	✓	Θ	Fourth-order FVM	Sigma	3500
Zmijanovic et al. [34]	✓	Θ	✓	Θ	Θ	Fourth-order FVM	Sigma	3500
Chabannes et al. [4]	✓	✓	✓	Θ	Θ	Taylor-Hood FEM	None	2000
Janiga [15]	Θ	Θ	Θ	Θ	✓	Low-order FVM	Smagorinsky	Θ
Passerini et al. [20]	✓	✓	✓	Θ	Θ	Low-order FEM	None	2000
Delorme et al. [6]	✓	✓	✓	✓	Θ	High-order FD, IBM	Vreman	2000
White and Chong [32]	✓	Θ	Θ	Θ	Θ	LBM	None	Θ

*Re*_*th*_ refers to the Reynolds number at the throat of the nozzle. The signs ✓and Θ respectively refer whether or not a particular *Re*_*th*_ was studied by the corresponding author group. *Re*_*crit*_ indicates the Reynolds number at which flow transitioned in the corresponding study. Part of the information is extracted from Fehn et al. [7]

The US Food and Drug Administration (FDA) established a benchmark in 1999 for this purpose [8]. This benchmark was developed to contain features that could closely resemble those in medical devices including regions of flow contraction and expansion, flow recirculation, local high shear stresses, and flow regimes ranging from laminar to transitional and turbulent. In addition, the benchmark was ensured to be simple enough such that CFD analyses could be readily performed and compared with experiments. The benchmark was thus a cylindrical pipe with a gradual contraction and sudden expansion. Particle image velocimetry (PIV) experiments were conducted by several laboratories on this device with Reynolds numbers 500, 2000, 3500, 5000, and 6500 to study all the flow regimes, namely laminar, transitional, and fully developed turbulence. A large interlaboratory variability was found in experiments especially in cases with transitional flow regimes (*Re*_*th*_ = 2000&3500) [26]. The experimental datasets were publicly released with the intention of serving as a benchmark for the validation of CFD solvers thereof.

Various numerical schemes ranging from finite differences to finite element and finite volume methods as well as high-order discontinuous Galerkin (DG) methods have been employed by researchers to evaluate if their numerical method and the corresponding implementation can reproduce the results published by the FDA. Table 1 lists major numerical studies conducted on the FDA benchmark and the Reynolds number (*Re*_*crit*_) at which the corresponding study found flow transition. In particular researchers evaluate if their CFD technique can predict the jet breakdown location and quantities like pressure and velocity accurately. Many studies explore parameters and appropriate mesh densities at which their results would match with the experimental data. Some of these studies have questioned the suitability of the FDA benchmark itself, inquiring if the benchmark should contain more information and be more robust for comparison with CFD. More interestingly some groups have found flow to transition to turbulence at *Re*_*th*_ = 2000 [4, 6, 20] whereas others have found flow transition only at *Re*_*th*_ = 3500 [1, 34].

The study of Fehn et al. [7] is the only exhaustive work to date in which authors applied a high-order DG method to study all the *Re*_*th*_, and even studied additional *Re*_*th*_ to explore the *Re*_*crit*_, i.e., the critical Reynolds number at which the flow would transition in the nozzle. Several others demonstrated different jet breakdown locations compared with experiments [1, 20], and some focused on outcomes like the *ever changing* location of jet breakdown with increasing spatial resolutions [1]. Furthermore, it is interesting to note that in some of the previous studies [34], adding synthetic fluctuations to the inflow was necessary to make numerical simulations agree with experiments for the *Re*_*th*_ = 3500 case.

The lattice Boltzmann method (LBM), which is an alternative and relatively new technique for the numerical solution of Navier-Stokes equations (NSE), has been extensively used in the past decade by fluid dynamics researchers, and it has found particular interest in the biomedical community [2, 13, 29, 33] as it can represent complex anatomical geometries with ease and can enable simulations on massively parallel computing architectures [13]. Recent works [12, 13] applied and validated LBM for complex transitional flows in anatomical geometries and found the method efficient and valid for such flow regimes. Despite pressing needs, however, no extensive effort to the author’s knowledge has been made to evaluate its efficacy in the aforesaid FDA nozzle benchmark. On the other hand, due to the diversity of results from different CFD studies and varied opinions about the suitability of the FDA benchmark, it becomes imperative to explore the suitability of the benchmark itself using a method that has not been applied to it before. White and Chong [32] did apply LBM to the FDA nozzle benchmark but only for the laminar case and their study was more oriented towards exploring the suitability of different lattice types. The previous works of transitional flow computations using LBM [12, 13] were also for moderate Reynolds numbers.

Motivated by the aforementioned needs, this work aims to evaluate if a simple LB scheme, without the employment of complex collision models or synthetic turbulence at the inflow, will accurately predict the results benchmarked by the FDA. The focus is on transitional flow regime and thus Reynolds numbers 2000 and 3500 are only analyzed. Physical quantities like *velocity, shear stress*, and *pressure* and observations like the *jet breakdown* location are compared from experiments and simulations. Furthermore, insight into questions like *when (Re)*, *where (locations)*, *whether*, and *how* of flow transition is provided. These goals are achieved by conducting LBM-based direct numerical simulations (DNS) at a very high, and another extreme resolution, which is found to be below the scales defined by Kolmogorov theory. A comparison against Kolmogorov theory and assessment of Kolmogorov scales is further shown to elucidate the role that resolutions play in computation of complex flow in such a medical device.

## Methods

### The FDA nozzle

**Fig. 1:**
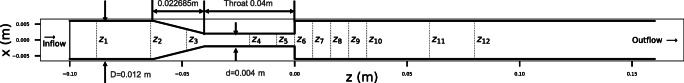
Cross-sectional view of the FDA nozzle for the sudden expansion case. Results were analyzed at various z-locations marked by dotted vertical lines to compare against FDA’s experimental results: *z*_1_ = − 0.088*m*,*z*_2_ = − 0.064*m*,*z*_3_ = − 0.048*m*,*z*_4_ = − 0.02*m*,*z*_5_ = − 0.008*m*,*z*_6_ = 0.0*m*,*z*_7_ = 0.008*m*,*z*_8_ = 0.016*m*,*z*_9_ = 0.024*m*,*z*_10_ = 0.032*m*,*z*_11_ = 0.06*m*,*z*_12_ = 0.08*m*

A cross-sectional view of the FDA nozzle is shown in Fig. 1. The flow direction is from left to right implying a sudden expansion. If the flow is applied from right to left, the same geometry will act as a canonical diffuser, which is not studied in this work. The 3D model of this nozzle was downloaded from the FDA website.^1^ The outlet of the model was extended up to *z* = 0.253 m and the inlet was extended up to *z* = − 0.14 m. The radial profiles were recorded at 12 locations depicted in Fig. 1 to enable a comparison against the particle image velocimetry (PIV) experimental data released by the FDA.

Simulations were conducted with three different spatial and temporal resolutions in order to explore the capabilities of an *off the shelf* LBM on a desktop machine to a federal supercomputer. Based on previous observations on resolution requirements in LBM simulations of transitional flow [14], a spatial resolution of about 80*μm* was predicted as a minimum requirement for simulation of *Re*_*th*_ = 2000 and accurate analysis of flow characteristics thereof. This resolution resulted in $\sim 45$ million lattice sites representing fluid inside the nozzle and 50 lattice cells across the height of the nozzle throat. This is referred to as normal resolution (NR) hereon. For *Re*_*th*_ = 3500 however, the flow was expected to transition and a high resolution (HR) of 40*μm* was thus chosen, which resulted in about 357 million lattice cells. As would be seen in Section 3, HR resolution sufficed for accurate flow simulations for both the Reynolds numbers. To further ensure mesh independence, assess the flow in accordance with Kolmogorov theory, and especially because it was not known whether LBM simulations will transition to turbulence, an additional set of simulations were conducted with an *extreme* resolution (XR) of 20*μm* resulting in about 2.88 billion lattice cells in the fluid domain.

Table 2 lists the employed spatial and temporal resolutions, the resulting number of lattice cells, the utilized CPUs, and total execution time of the simulation.

**Table 2:** Three different spatial and temporal resolutions and the corresponding number of lattice cells inside the fluid domain

	*δx*(×10^− 6^*m*)	*δt*(×10^− 6^*s*)	nCells_th_	nCells(M)	nCores	*T*(h)
NR	80	16	50	45	32	192
HR	40	4	100	357	38^′^016	3.5
XR	20	1	200	2^′^880	304^′^128	12

The NR simulations were conducted on 32 cores of a desktop while others on the *SuperMUC-NG*. A total of 10 physical seconds were simulated for all the resolutions

### Setup of a simulation using the lattice Boltzmann method

To shed light on the aforementioned choice of resolutions and LB parameters, a brief explanation is provided here. The LBM is based on the mesoscopic representation of movement of fictitious particles. The particles have discrete velocities and they collide and stream to relax towards a thermodynamic equilibrium. The LB equation recovers the NSE under the continuum limits of low Mach and Knudsen numbers. Evolution of the particle probability distribution functions over time is described by the lattice Boltzmann equation with the MRT collision matrix:

1 $$ \begin{array}{@{}rcl@{}} f_{i}(\mathbf{r} + \mathbf{c}_{i}\delta t, t + \delta t) = f_{i}(\mathbf r,t) + {\Omega}_{ij} \left( {f_{i}^{e}}(\mathbf r,t) - f_{i}(\mathbf r,t) \right) \end{array} $$

where *f*_*i*_ represents the density distributions of particles which are moving with discrete velocity **c**_*i*_ at a position **r** at time *t*. The indices which run from *i* = 1 … *Q* denote the links per element, i.e., the discrete directions, depending on the chosen stencil (D3Q19 in our case). The collision matrix Ω_*ij*_ defines relaxation of various modes of the distribution functions *f*_*i*_ towards an equilibrium ${f_{i}^{e}}$:

2 $$ \begin{array}{@{}rcl@{}} {f_{i}^{e}} = w_{i} \rho \Bigg(1 + \frac{\mathbf{c}_{i} \cdot \mathbf{u}}{{c_{s}^{2}}} - \frac{\mathbf{u}^{2}}{2{c_{s}^{2}}} + \frac{1}{2} \frac{(\mathbf{c}_{i} \cdot \mathbf{u})^{2}}{{c_{s}^{4}}} \Bigg) \end{array} $$

where *w*_*i*_ are the weights for each discrete link, *c*_*s*_ is the reference speed of sound in LBM obtained by integration of the discrete Boltzmann equation along characteristics, and **u** is the fluid velocity. The time step in LBM is coupled with the grid size by $\delta t \sim \delta x^{2}$ due to *diffusive* scaling which is employed to recover the incompressible NSE. Details on the computation of macroscopic quantities from LBM can be found elsewhere [28]; here we restrict ourselves on the process of setting up a flow simulation.

#### Prescription of flow physics

Quantities like the mean velocity, $\bar {u}$, on which the Reynolds number is based, fluid density *ρ*, and the kinematic viscosity *ν* are firstly chosen for the fluid to be simulated. The Reynolds number is then calculated as:

3 $$ \begin{array}{@{}rcl@{}} \text{Re} = \frac{\bar{u}  d}{\nu} \end{array} $$

where *d* is the characteristic length equal to the throat diameter (0.004m) in this case. In this study, the Reynolds number is based on the nozzle throat.

The LB method requires the translation of these quantities into lattice units. Under diffusive scaling, the principal relaxation parameter Ω is fixed and can have a maximum value of 2.0 for stability. The lattice viscosity, *ν*_*lattice*_, is then calculated using the relation:

4 $$ \begin{array}{@{}rcl@{}} \nu_{lattice} = {c_{s}^{2}} \bigg(\frac{1}{\Omega} - \frac{1}{2} \bigg) \end{array} $$

where ${c_{s}^{2}}$ is the speed of sound squared at reference state and is equal to $\frac {1}{3}$ for the chosen stencil D3Q19. The time step is then calculated as:

5 $$ \begin{array}{@{}rcl@{}} \delta t = \frac{\nu_{lattice}  \delta x^{2}}{\nu} \end{array} $$

Finally, the lattice velocity is obtained as:

6 $$ \begin{array}{@{}rcl@{}} \bar{u}_{lattice} = \bar{u}  \delta t / \delta x \end{array} $$

The MRT collision operator allows for different relaxation rates of various moments of the distribution function, and it was employed throughout for the DNS reported in this manuscript. The principal relaxation parameter that defines the kinematic viscosity was set to Ω = 1.90. It is required that the $\bar {u}_{lattice}$ in Eq. 6 is always less than 0.15 to enforce the Mach number limit of the LBM. The Ω can be adjusted in order to fine tune the lattice velocity and beyond its limit, the grid has to be refined (reduce the *δx*), which is also the principle limitation of the lattice Boltzmann method. Thus, the *δt* is controlled by the *δx* and the Ω, and it is further constrained by the fluid velocity. To assert correct prescription of parameters, if the characteristic length in Eq. 3 is replaced by the number of fluid cells along that particular characteristic length and $\bar {u}$ and *ν* are replaced by $\bar {u}_{lattice}$ and *ν*_*lattice*_, respectively, the same Reynolds number must be obtained as that obtained from Eq. 3, which uses physical values.

#### The simulation framework

The employed simulation tool-chain is contained in the end-to-end parallel framework APES (adaptable poly engineering simulator) [17, 24].^2^ Meshes are created using the mesh generator *Seeder* [9] and computations are carried out using the LBM solver *Musubi* [10]. *Musubi* writes out binary files containing physics information to the disk. These files are then converted to the visualization toolkit (VTK) format by the post-processing tool *Harvester*, which is contained within the APES framework. The open-source visualization tool Paraview^3^ is then used to visualize the physics of flow. The data for plots is written out by *Musubi* as ASCII files that are plotted using the Matplotlib plotting library within the Python programming language.

The 3D model of the nozzle in STL format was read by the mesh generator *Seeder* and volume meshes for LBM computations were saved on the disk. A higher order wall boundary condition described by Bouzidi et al. [3] was prescribed at the walls of the nozzle to reduce the influence of staircase artifacts in LBM and ensure rotational symmetry of the setup. The *Musubi* LBM solver then computed these meshes (see Table 2) on the *SuperMUC-NG* petascale system installed at the Leibniz Supercomputing Center in Munich, Germany. The number of utilized cores on *SuperMUC-NG* ranged from 38,016 to 304,128 (the whole system) based on our previous evaluations which have shown that this choice of CPUs results in minimal communication overhead among CPUs thereby ensuring the most optimal utilization of compute resources [17, 23].

#### Initial and boundary conditions

Initial conditions were set to zero pressure and velocity and a no-slip boundary condition was maintained at the walls. The flow rates at inlet were characterized based on the throat Reynolds number. The fluid itself was chosen to be Newtonian, with a fluid density of 1056*kg*/*m*^3^ and dynamic viscosity of 0.0035*Ns*/*m*^2^.

A parabolic velocity profile was prescribed at the inlet by quadratically interpolating the velocity at lattice nodes, and a zero pressure was maintained at the outlet. No synthetic turbulence was prescribed at the inlet unlike some previous studies. The outflow in such flow regimes can be critical resulting in back flow or instabilities in the LBM algorithm for which an extrapolation boundary condition was employed [16].

Following Fehn et al. [7], the simulated time interval was chosen on the basis of a characteristic flow-through time. The length of the throat, *L*_*th*_, section and the mean velocity, $\bar {u}$, were chosen as characteristic length scale and the reference velocity respectively. This resulted in a flow through time given by:

7 $$ \begin{array}{@{}rcl@{}} T_{ft} = \frac{L_{th}}{\bar{u}} \end{array} $$

For the *Re*_*th*_ = 2000 case, this time was approximately 0.21 s whereas for the *Re*_*th*_ = 3500 case, it was 0.12 s. Flow was allowed to develop for initial 2 s implying about 10 and 20 flow throughs for the *Re*_*th*_ = 2000 and *Re*_*th*_ = 3500 case respectively. Within the computational bounds, a total of 10 physical seconds were simulated for each case. A total of 50,000 samples were gathered every second for the analysis of flow characteristics.

### Flow characterization

For the analysis of turbulent characteristics, the three-dimensional velocity field was decomposed into a mean and a fluctuating component as:

8 $$ \begin{array}{@{}rcl@{}} u_{i}(x,t) = \bar{u}_{i}(x) + u_{i}^{\prime}(x,t) \end{array} $$

The turbulent kinetic energy (TKE) was derived from the fluctuating components of the velocity in three directions as:

9 $$ \begin{array}{@{}rcl@{}} k = \frac{1}{2}\Big({u_{x}^{\prime 2} + u_{y}^{\prime 2} + u_{z}^{\prime 2}} \Big) \end{array} $$

The dimensionless Strouhal number, *St*, is defined as:

10 $$ \begin{array}{@{}rcl@{}} St = \frac{f d}{\bar{u}} \end{array} $$

where *f* is the frequency of flow fluctuations and *d* and $\bar {u}$ are the characteristic length and mean velocities. A power spectral density of the TKE was computed as a function of the Strouhal number at various centerline locations using the Welch’s periodogram method to analyze the inertial and viscous subranges.

### Kolmogorov microscales

The smallest structures that can exist in a turbulent flow can be estimated on the basis of Kolmogorov’s theory [22]—used in this work to assess the quality of the resolutions in DNS. Viscosity dominates and the TKE is dissipated into heat at the Kolmogorov scale [22]. The Kolmogorov theory, in general, applies to fully developed turbulent flows with Reynolds numbers much higher than those studied in this work. The reference to Kolmogorov scales and theory here is thus indicative for the assessment of mesh independence as has been done in various such studies at low Reynolds numbers [11, 12].

The Kolmogorov scales, non-dimensionalized with respect to the velocity scale $\bar {u}$, and the length scale *d* (throat diameter) are computed from the *fluctuating* component of the non-dimensional strain rate defined as:

11 $$ \begin{array}{@{}rcl@{}} s^{\prime}_{ij} = \frac{1}{2}\bigg(\frac{\partial u_{i}^{\prime}}{\partial x_{j}} + \frac{\partial u_{j}^{\prime}}{\partial x_{i}}\bigg) \end{array} $$

The Kolmogorov length, time, and velocity scales are then respectively computed as:

12 $$ \begin{array}{@{}rcl@{}} \eta = \bigg(\frac{1}{Re^{2}} \frac{1}{2 s^{\prime}_{ij}s^{\prime}_{ij}} \bigg)^{1/4} \end{array} $$

13 $$ \begin{array}{@{}rcl@{}} \tau_{\eta} = \bigg(\frac{1}{2 s^{\prime}_{ij}s^{\prime}_{ij}}\bigg)^{1/2} \end{array} $$

14 $$ \begin{array}{@{}rcl@{}} u_{\eta} = \bigg(\frac{2s^{\prime}_{ij}s^{\prime}_{ij}}{Re^{2}} \bigg)^{1/4} \end{array} $$

Based on these scales, the quality of the spatial and temporal resolutions employed in a simulation can be estimated as the ratio of *δx* and *δt* against the corresponding Kolmogorov scales, i.e.,

15 $$ \begin{array}{@{}rcl@{}} l^{+} = \frac{\delta x}{\eta}  t^{+} = \frac{\delta t}{\tau_{\eta}} \end{array} $$

The Kolmogorov scales in this study were computed along various locations along the centerline. The fluctuating strain rate was averaged between locations *z*_8_ and *z*_12_ (Fig. 1) due to variations in TKE caused by jet breakdown in these locations, and the Kolmogorov scales were computed from this averaged strain rate.

**Table 3:** Kolmogorov scales computed from NR, HR, and XR resolutions for *Re*_*th*_ = 2000 and *Re*_*th*_ = 3500

	*Re*_*th*_ = 2000					*Re*_*th*_ = 3500				
	*η*(*μm*)	*τ*_*η*_(*μs*)	*u*_*η*_(*m*/*s*)	*l*^+^	*t*^+^	*η*(*μm*)	*τ*_*η*_(*μs*)	*u*_*η*_(*m*/*s*)	*l*^+^	*t*^+^
NR	4.94	8.37	1.19	16.19	1.91	Θ	Θ	Θ	Θ	Θ
HR	16.32	8.69	1.97	2.45	0.46	12.57	4.08	3.80	3.18	0.98
XR	21.97	7.14	2.10	0.91	0.14	18.86	4.34	4.10	1.06	0.23

### Comparison with experiments

The radial velocity profiles in the streamwise direction and the mean centerline velocity at 12 locations shown in Fig. 1 were plotted against corresponding data from 5 PIV experiments. Instantaneous centerline velocities and pressure were analyzed at 120 locations each 0.002 m apart along the length of the nozzle. The 5 FDA experiments were conducted by 3 independent laboratories where one laboratory ran three trials resulting in 5 data sets [8]. The axial component of the velocity *u*_*z*_ was plotted directly against the data from experiments.^4^

Pressure was probed and averaged at 12 circular cross sections across the length of the nozzle (*z*_1_ − *z*_12_). To enable comparison against experiments, the mean pressure difference was normalized with respect to the average velocity at the throat $\bar {u}$:

16 $$ \begin{array}{@{}rcl@{}} {\Delta} p^{norm} = \frac{p_{z} - p_{z_{0}}}{0.5 \rho \bar{u}^{2}} \end{array} $$

where *ρ* is the fluid density and $\bar {u}$ is the mean velocity at the nozzle throat, on which the *Re*_*th*_ was based (see Eq. 3).

The shear stress was computed from the LBM simulations as it is one of the quantities of interest to predict hemolysis, and was directly compared with the experimental data.

The differences between a particular LBM simulation case and an experimental dataset were quantified on the basis of simple relative percentage errors, computed as:

17 $$ \begin{array}{@{}rcl@{}} \delta = \left|~\frac{U_{ref} - U_{h}}{U_{ref}}~\right| \times 100 \end{array} $$

where *U*_*ref*_ denotes the reference solution (experiments in our case) and *U*_*h*_ denotes the LBM computed solution; the corresponding solutions can be for pressure, velocity, or shear stress.

## Results

The flow transitioned to turbulence at *Re*_*th*_ = 2000 and it became fully turbulent at *Re*_*th*_ = 3500. The NR simulation was stable for *Re*_*th*_ = 2000 during the whole 10 s that were simulated. For *Re*_*th*_ = 3500, however, the NR simulation crashed as soon as the flow reached the outflow due to the instabilities in that region, and local fluctuations in the Mach number limit of the LB algorithm (6).

### Kolmogorov microscales

To assess the mesh independence of the simulations, we start with an analysis of the Kolmogorov microscales, shown for different resolutions and *Re*_*th*_ in Table 3.

The *l*^+^ from NR resolution for *Re*_*th*_ = 2000 is 16.19 thus clearly indicating this resolution as insufficient for accurate assessment of turbulent characteristics. These scales from the HR simulations for both *Re*_*th*_ are sufficiently close to the corresponding Kolmogorov scales while from XR simulations they attain a value equal to the Kolmogorov scales. As observed later, the XR resolutions, being resolved exactly at the Kolmogorov length scales, result in minor deviances in flow characteristics if compared with HR resolutions. The *τ*_*η*_ are < 1 in most of the cases due to the small time step of LB simulations.

### Jet breakdown location

**Fig. 2:**
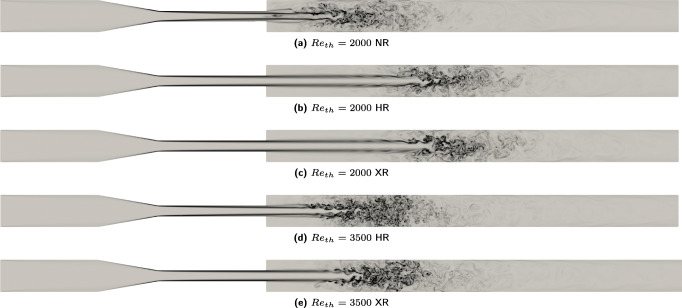
Snapshots of the instantaneous vorticity during *t* = 10 second of the simulation across a bisecting plane in the FDA nozzle from HR and XR LBM simulations for *Re*_*th*_ = 2000 and *Re*_*th*_ = 3500. The vorticity magnitude is scaled according to *Re*_*th*_ and ranges from 0 to 2*Re*_*th*_

The regions of flow breakdown and maximum turbulent activity are highlighted in Fig. 2, which shows the instantaneous vorticity magnitude at the *t* = 10 second of simulations. Jet breakdown location identified from NR simulations of *Re*_*th*_ = 2000 lies between *z*_9_ and *z*_10_. A secondary flow jet however starts emanating right after the expansion. It may be concluded that the NR resolution captures the onset of turbulence but with a largely compromised accuracy as the location of jet breakdown is different from all the previously reported experiments [26]. On the other hand, the vorticity plots for *Re*_*th*_ = 2000 from both HR and XR LBM simulations demonstrate that the location of jet breakdown is more or less identical (between *z*_11_ and *z*_12_) and resolutions do not seem to influence the location of jet breakdown. The jet breakdown location is between *z*_9_ and *z*_10_ at *Re*_*th*_ = 3500 from HR simulations while it is shifted further downstream by approximately half of the throat diameter from XR simulations. It is emphasized that these are *instantaneous* vorticity plots during the *t* = 10 second of the simulation as due to immense memory requirements an ensemble averaging was not possible. Furthermore, it is expected that an ensemble average of at least 10,000 snapshots would be needed to have an accurate assessment of jet breakdown location.

In the Supplementary Material, animations of vorticity and velocity field for both *Re*_*th*_ from HR simulations are provided over the last one second (9 − 10) of the simulations. It may be observed from the animations that at *Re*_*th*_ = 2000 the jet itself experiences distortion over time course of the simulation. If we take a closer look at Fig. 2b, we can see development of *discontinuities* over the jet after 3 nozzle diameters downstream of expansion. When the flow breaks down, vortices merge, annihilate, and interact with the jet to *distort* the jet itself over the course of time and resolution does not seem to play a role here. This circumstance is in fact natural for a transitional flow as it is not fully developed turbulence but the jet is between laminar and transitional regime. For *Re*_*th*_ = 3500, on the other hand, the jet does not get distorted over the course of time but it shifts further downstream at XR resolution (Fig. 2e). In this case, a shear layer develops around the jet itself as can be clearly seen from the animations as well as the instantaneous snapshots of vorticity. A further grid refinement for the case of *Re*_*th*_ = 3500 (Fig. 2e) resolves this shear layer better to shift the jet breakdown location further downstream by about half a throat diameter (*d*/2 = 0.002*m*) as this is a fully developed turbulent flow field.

**Fig. 3:**
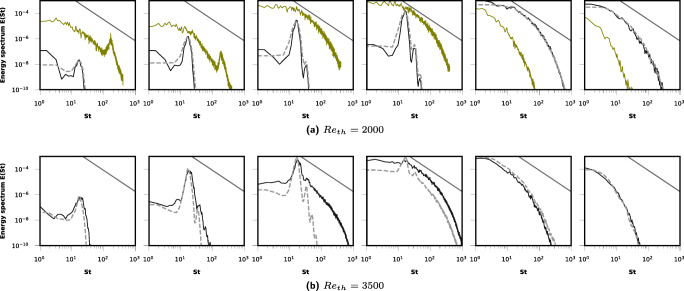
Spectral density of the turbulent kinetic energy at 6 locations along the centerline after the sudden expansion (*z*_7_ − *z*_12_ in Fig. 1) along the centerline is plotted against the Strouhal number (St). The PSD is computed during the last 8 s of the simulations using 4 × 10^5^ time steps in total. Dark and dotted gray lines show the PSD respectively from HR and XR LBM simulations. For *Re*_*th*_ = 2000, PSD from NR simulations is shown in green line. The solid gray line shows Kolmogorov’s $\frac {-5}{3}$ decay

**Fig. 4:**
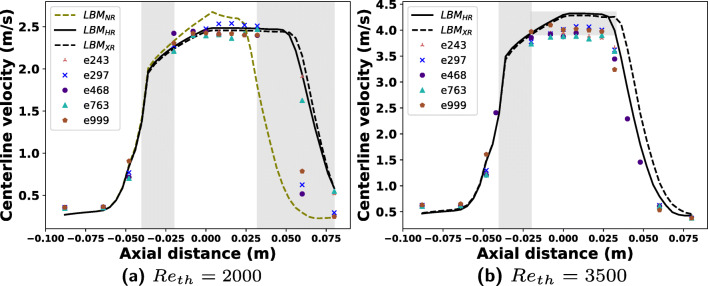
Time averaged centerline velocity at 86 locations along the length of the nozzle for two different Reynolds numbers and all the employed LBM resolutions. The centerline velocity is compared with 12 (*z*_1_ – *z*_12_ from Fig. 1) experimental data points along the centerline available from 5 distinct PIV experiments. The number in the legend corresponds to the experiment ID from the FDA website. The gray bars highlight the regions with largest discrepancy between experiments and simulations. At *Re*_*th*_ = 3500, the NR simulation was unsuccessful

An accurate assessment of turbulent activity at various locations, and from different resolutions, can be obtained from the PSD plots of Fig. 3, computed at locations downstream of the expansion (*z*_7_ − *z*_12_ from Fig. 1). The PSD is computed over 8 seconds of the simulation to obtain abundant statistics and overcome previous issues of insufficient averaging. The dark and dotted gray lines respectively show PSD from HR and XR LBM simulations whereas the solid gray line indicates Kolmogorov’s $\frac {-5}{3}$ decay. For *Re*_*th*_ = 2000, the green line shows the PSD from NR simulations, which corroborates previous observations from vorticity plots and Kolmogorov scales and confirms the inadequacy of this resolution. For the *Re*_*th*_ = 2000 case, it is seen that the flow transitions at locations *z*_11_ and *z*_12_ and the turbulent activity captured by HR and XR simulations is the same with a few frequencies in the inertial subrange. The third plot of Fig. 3b is the most enlightening about the jet breakdown location for *Re*_*th*_ = 3500 from HR and XR simulations and corroborates the observations from plot Fig. 4b. Clearly the jet does not break down at location *z*_9_ in XR simulations as the spectrum falls off rapidly whereas in HR simulations the spectrum tends to attain a Kolmogorov like decay [12] at this location. The following figures then substantiate this observation whereby at *z*_10_ the jet is already turbulent from both HR and XR simulations and turbulent activity becomes similar beyond this (last two plots of Fig. 3b).

### Comparison of velocity, pressure, and shear stress against experiments

Figure 4 shows the averaged centerline velocity computed at 86 axial locations between *z*_1_ and *z*_12_ for both the Reynolds numbers from HR and XR LBM simulations and additionally from NR simulations for the *Re*_*th*_ = 2000 case. The corresponding data from 5 experiments at 12 locations is plotted for comparison. The shaded bars in the plots highlight the regions of largest discrepancy between experiments and simulations. As can be seen, for *Re*_*th*_ = 2000, there is a significant difference between experiments and simulations between locations *z* = − 0.04 and *z* = − 0.02, which is the region just after the end of gradual contraction, or the beginning of the nozzle throat. The velocity matches well at the latter part of the throat and this difference is seen again in the jet breakdown location, which matches reasonably with one experiment only (*e*243) for both the HR and XR resolutions. The difference in velocity in regions between *z* = 0.06 and *z* = 0.07 is about 10*%* between few experiments and simulations, computed using (17). From NR simulations, the jet breakdown location is completely different from experiments and HR and XR simulations as was seen in previous plots.

For the *Re*_*th*_ = 3500 case, similar trends in the throat area are seen while the jet breakdown location from HR simulations matches more closely to the experiments. This location from the XR simulations is comparatively further away by half the throat diameter. The velocities estimated by the LBM at the throat are also higher by about 6–9*%* than those from the experiments. For *Re*_*th*_ = 3500, the velocities computed by LBM right after the expansion at locations around *z* = 0.025 are overestimated by about 10*%* compared with the experiments.

The cause of discrepancies in the starting of the throat area (shaded regions) cannot be unequivocally stated as the data points available from the experiments are very few. In the plot from the simulations, there are 14 samples between locations *z*_3_ and *z*_4_ (Fig. 1). If the data points between these two locations were omitted, the curves would look like a straight line as in the experiments and previously reported studies [7]. The pressure at the throat right after the contraction reduces considerably and the minor discontinuities at the corners might explain these differences.

**Fig. 5:**
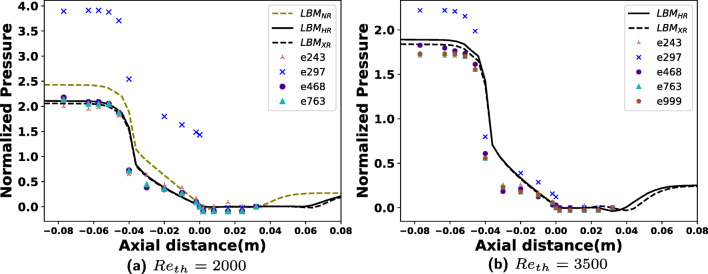
Time averaged centerline pressure normalized by the mean throat velocity *u*_*th*_ versus the axial distance computed from two sets of LBM resolutions is compared against pressure data from 5 PIV experiments of the FDA for two different Reynolds numbers. Experimental data is plotted at 12 locations along the centerline (*z*_1_ – *z*_12_ in Fig. 1) for experiments whereas 86 points between these locations are plotted from simulations. Note that for *Re*_*th*_ = 2000 case, the pressure data from experiment *#*999 is not available. At *Re*_*th*_ = 3500, the NR simulation was unsuccessful

This is further elaborated in the centerline average pressures plotted in Fig. 5. Qualitatively, similar trends from experiments and simulations are observed, and the difference between HR and XR simulations for both the Reynolds numbers is much smaller. Interestingly, the pressure drop for *Re*_*th*_ = 3500 at locations before the expansion (*z* < 0.0) is overestimated by about 5–6*%* and matches only one experiment. The data points for pressure from PIV experiments are relatively few disabling a better and insightful comparison. Furthermore because the outflow length in LBM simulations is larger than that in experiments, it may account for minor differences in pressure changes.

**Fig. 6:**
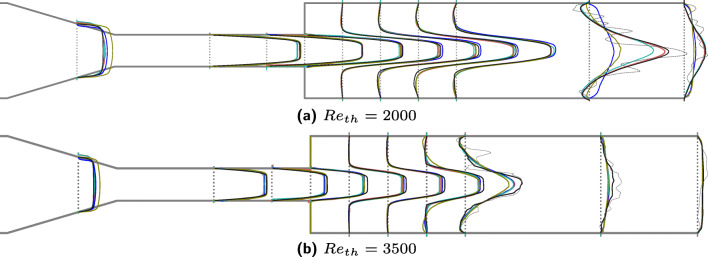
Radial velocity profiles at 10 locations (*z*_3_ − *z*_12_ shown in Fig. 1) along the length of the nozzle for two different Reynolds numbers averaged over 8 s or 400,000 time steps. The velocities are normalized to enable an intuitive comparison. The black lines show the averaged velocities computed from XR LBM simulations whereas red, blue, green, and olive-colored lines are the data from experiment ids *#*243, *#*297, *#*763, and *#*999 respectively. The gray dotted lines at locations *z*_11_ and *z*_12_ for *Re*_*th*_ = 2000 and at *z*_10_, *z*_11_, and *z*_12_ for *Re*_*th*_ = 3500 show instantaneous radial velocities from LBM simulations to depict the location of jet breakdown

Figure 6 shows the radial profiles of mean streamwise velocity from XR LBM simulations at 10 locations after the contraction (*z*_3_ − *z*_12_ from Fig. 1). The ensemble averages are gathered over the last 8 s of the simulation or for 4 × 10^5^ time steps. In this case, the velocities have been normalized by $\bar {u}$ for a direct intuition about the differences in experiments and simulations. The red-, blue-, green-, and olive-colored lines are the data from experiment ids *#*243, *#*297, *#*763, and *#*999 respectively.^5^ At locations *z*_11_ and *z*_12_ for *Re*_*th*_ = 2000 and at *z*_10_ − *z*_12_ for *Re*_*th*_ = 3500, the instantaneous radial profiles at *t* = 10 second of the simulation are plotted in gray dotted lines to depict regions where the flow jet broke down. For the *Re*_*th*_ = 3500 case, the velocity from LBM in the post expansion region is higher than the experiments mainly in the jet core region as was also observed in Fig. 4b. Except for the jet breakdown locations, a reasonable agreement in radial profiles can be seen against all the experiments. As has been observed throughout this study, the interlaboratory variations in the experiments are quite large [26] making a quantitative comparison more difficult.

**Fig. 7:**
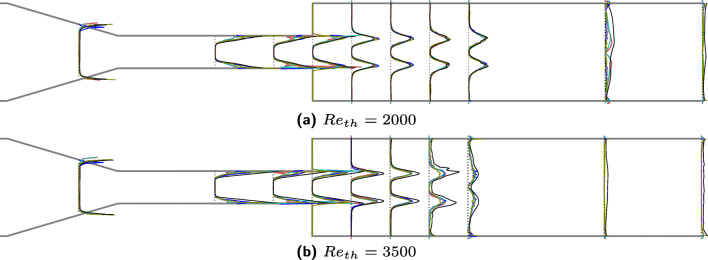
Radial shear stress profiles at 10 locations (*z*_3_ − *z*_12_ shown in Fig. 1) along the length of the nozzle for two different Reynolds numbers averaged over 8 s or 400,000 time steps. The shear stresses are normalized to enable an intuitive comparison. The black lines show the averaged velocities computed from XR LBM simulations whereas red, blue, green, and olive-colored lines are the data from experiment ids *#*243, *#*297, *#*763, and *#*999 respectively

Shear stress from experiments and computations is compared in Fig. 7. Here the shear stress has been normalized by mean shear stress so that a direct comparison can be enabled. Excellent agreement for both the Reynolds numbers can be observed in mean radial velocities as well as the shear stress.^6^ The shape of the shear stress profile at locations before the expansion (*z* < 0.0) is flatter in the simulations whereas in experiments it exhibits a skewed shape. Other minor differences lie in the regions of jet breakdown and are a consequence of the fact that 4 × 10^5^ sample points are used for gathering statistics, while good, are not perfect for the reproduction of a converged state. Despite the computational resources deployed for this study, sampling more statistics was considered prohibitive. For *Re*_*th*_ = 3500, LB overestimates the velocity and the shear stress at the location of jet breakdown, which was also observed in Fig. 4. These differences are about 3–4*%* are mostly confined around the jet breakdown locations.

## Discussion

It is the first time that LBM has been applied to study transitional flows in the two-decade-old FDA benchmark. The study has found that LBM can predict the jet breakdown location and compute macroscopic quantities to a reasonable accuracy for a transitional flow in a biomedically relevant device. In addition to a comparison, the main physical insight is the distortion in jet for transitional flow case of *Re*_*th*_ = 2000 and assessment of the Kolmogorov microscales. Here we discuss the agreement and discrepancy in results as well as implications of this study.

### Analysis of the flow characteristics and comparison with previous works and experiments

Qualitatively the LB computed flow transitioned to turbulence at *Re*_*th*_ = 2000 as it did in most of the experiments [26], and flow field at *Re*_*th*_ = 3500 was reminiscent of a partially developed turbulence indicated by the immense chaotic activity. Quantitatively, a satisfactory match between experiments and computations was observed in averaged velocities, shear stress, and pressure as well as the jet breakdown locations. This agreement has been obtained without parameter tuning in the method, and in particular without adding synthetic fluctuations as has been necessary for other studies [34]. The centerline velocity and pressures computed from LBM had marked differences, in particular in the throat and the expansion area. In particular for the *Re*_*th*_ = 3500 case, the LB computed velocities were higher by about 10*%* in comparison with the experiments. While an exact reason for this is not known, it may be observed that the LB computed radial velocity profiles (Fig. 6b) had a more pronounced recirculation region and a higher velocity only in the jet core—not seen in the experiments.

A mesh sensitivity analysis revealed that resolutions within an order of magnitude of the Kolmogorov microscales are necessary to accurately capture the flow field and a further refinement down to the Kolmogorov scales results in slightly distal jet breakdown of flow downstream of expansion for *Re*_*th*_ = 3500 while the averaged macroscopic quantities are not much affected. The NR resolution failed for the higher *Re*_*th*_ = 3500 and the results were erroneous for *Re*_*th*_ = 2000. Previous studies have found a much larger dependence of results on the mesh density. Delorme et al. [6] used LES model and found good match except that their simulations found relatively less turbulent nature of the flow attributed to grid resolutions. Passerini et al. [20] performed a number of simulations for each *Re* they studied (Table 1) to arrive at an optimal grid resolution. Zmijanovic et al. [34] used three mesh resolutions in their LES simulations and found excellent match with experiments from the highest resolution of 50*M* cells. In the present LBM simulations, going from HR to XR increased the computational effort remarkably and brought the resolutions right down to the Kolmogorov microscales. The benefit of this was pronounced at *Re*_*th*_ = 3500 while at *Re*_*th*_ = 2000 almost no advantage in the results was seen from HR to XR. The HR setup is thus adequate in this configuration as suggested by previous studies [14] as well. The scalability of LBM to XR scale may be leveraged in future to incorporate physics of red blood cells or other particles in the blood [29] to answer relevant questions in physiology.

In the present work, the role of boundary conditions has not been explored in detail and the prescription of inflow and outflow boundary conditions is likely to influence the results. Passerini et al. [20] extended the in- and outflow according to the *Re*_*th*_ and the recent precursor approach adopted by Fehn et al. [7] seems to overcome most of the effects of inflow pipe length. When a simulation is refined, the accuracy of the boundary conditions also increases, which explains the *eternal* change in solution upon increasing resolutions. In particular, the disturbances that emanate from the outflow are reduced upon refining the mesh and time step, which would explain the relatively further breakdown of jet downstream of the expansion.

### Jet breakdown location

A number of previous studies have found the jet breakdown location sensitive to parameters used in CFD [20, 34]. Here, as noted above, there were no parameters that were varied in the LBM simulations except for the grid refinement and the corresponding time step size. For *Re*_*th*_ = 2000, we noted that the jet breakdown location was largely the same from HR and XR resolutions. A new observation, seemingly overlooked in previous studies, was the propagating distortion in the jet at *Re*_*th*_ = 2000 (animations in Supplementary Information). As the flow was just transitioning, the jet lost its momentum at times, tended to restabilize the flow, and then gained momentum again resulting in shifts in the breakdown location. For *Re*_*th*_ = 3500, the jet broke down half a nozzle diameter further downstream of the expansion in XR simulations compared with HR. The reason for that is the better accuracy at the sudden expansion, which stabilized the perturbances in that area, and their propagation thereof. Nonetheless, the breakdown location from XR simulations was just half the throat diameter, *d*/2, downstream, and its relevance can be queried. More interestingly the flow restabilization regions from both the resolutions were the same for *Re*_*th*_ = 3500, and the XR resolution thus narrowed down the length of chaotic activity (Fig. 3). It would be biased and impractical to extrapolate these observations of jet breakdown location to other numerical studies [7, 20, 34] as the LBM essentially solves the Boltzmann equation to recover NSE. Also, the parameters that have been of discussion in related studies [6, 7] like Courant number cannot be directly related with LBM. It is however important to remark that numerical dissipation in LBM, even at the scales of grid spacing, and the numerical dispersive effects are smaller compared with other second-order accurate methods [18], which to a certain extent explains the consistent jet breakdown locations with increasing resolutions.

In this study, we upsurged from NR to HR and XR directly without exploring a mid resolution range. It is noted that these are not a minimum resolution requirement for LBM simulations, and it is thus re-emphasized that this study was designed to explore LBM’s suitability in performing fully resolved DNS, and thus resolutions, *as high as possible*, within the confines of present computational paradigms were employed. It may be noted that the LB literature has grown considerably in the past and several ways to incorporate turbulence models within LB have been devised [27]. Evaluations of these models using the FDA nozzle are left for future efforts.

### Onset of flow transition

If we focus on the FDA nozzle only, previous studies have found conflicting *Re*_*crit*_, and why the numerically computed flow field did, or did not, transition at *Re*_*th*_ = 2000 in one particular study or the other is still unknown. It is fair to state that a flow in a perfectly symmetric setup as that of the FDA benchmark should not transition to turbulence in a simulation, and such an event must be a consequence of the numerics. Different numerical methods, parameters, stabilization techniques, and resolutions may thus result in suppression or amplification of a turbulence triggering mechanism. Intense discussions have curtailed in the past about the role of resolutions to capture transitional phenomena [6, 14, 31, 34] while the physics, non-linear dynamics of a transitional flow, and its mechanobiological significance, if any, must be viewed with equal attention. A closer look at Fig. 2b and the animations shows disturbance in the flow jet that emanates from the nozzle throat discussed above. These disturbances in the jet were also seen in the work of Fehn et al. [7] despite the flow remained laminar in their simulations at this *Re*_*th*_. Upon slight increase in *Re*_*th*_ to $\sim 2400$, the jet did breakdown in their simulations to transition the flow. It may be inferred that at *Re*_*th*_ = 2000, the flow is on the verge of breakdown, which, depending on the numerics and inflow conditions used, may or may not quantitatively breakdown. A complementary question is the circumstances that perturb the flow in the first place to trigger turbulence. It may be hypothesized that the perfect symmetry of the mesh in higher order methods suppresses the onset of transition but a conclusive statement on that cannot be made. Despite the fact that symmetry was ensured in the LB setup, there could have been artifacts from the boundaries that manifested as instabilities in the flow thereby triggering turbulence. White and Chong [32] demonstrated the inferior rotational invariance of the *D*3*Q*19 lattice and found *D*3*Q*27 superior whereas Dellar [5] advocated that multi-time relaxation (MRT) model of the LBM recovers the rotational invariance. Peng et al. [21] also recently found both these types of lattice to yield accurate turbulent flow statistics. None of the study to the author’s knowledge has investigated the role of lattice types in combination with the higher order wall boundary condition that was employed in this study [3] and also none has used these high resolutions. It may be inferred that a combination of MRT, higher order wall representation, and high resolutions must have overcome the minor deficiencies of the *D*3*Q*19 lattice but a detailed investigation of that is left for future efforts. It is also emphasized that the LBM is inherently a transient scheme, which might explain a closer match to the experiments. In a setup like the FDA nozzle, it is clear that the sudden expansion resulted in adverse gradients of pressure, which, at a sufficiently high *Re*_*th*_ departed the flow from its laminar regime.

It is finally remarked that the FDA nozzle is essentially a device to study blood flow and the non-Newtonian affects due to blood cells should be accounted for in future [25]. In LBM simulations, non-Newtonian models that account for shear thinning behavior of fluid can be easily incorporated, and such a model is expected to *delay* the onset of flow transition. On the other hand, however, Tupin et al. [30] have recently demonstrated a unique inverse energy cascade in blood flow and have found the turbulence of non-Kolmogorov type. The studies of transitional bioflows in future may study explicit transport of red blood cells, which requires incorporation of, and coupling with, other methods with LBM [29]. The present work has prospects to serve as reference for future extensions and comparisons.

## Conclusions and implications

1. The LBM is an adequate numerical technique for the DNS of biomedical flows in transitional regime, and can reproduce flow characteristics without much parameter tuning even at higher Reynolds numbers. The FDA benchmark, for transitional and turbulent flow regimes, is suitable but not fully vigorous for a detailed quantitative comparison of CFD codes. The definition of the benchmark should have a reliable and quantifiable turbulence triggering mechanism, which may be incorporated into CFD models.
2. The practice of *quantitatively* and *qualitatively* comparing experiments and simulations for a transitional flow is not entirely appropriate. Previously conducted experiments can only provide guidance on the setup of simulation and help in the analysis of results thereof. A comparison can only be performed by extensive joint efforts of experimentalists and computational researchers to *tweak* experimental aspects like noise at inflow according to the simulations, or adjusting simulation aspects like initial conditions or inflow distortions. Previously reported discrepancies between experimental and computational results are non-quantifiable and their source, while can be conjectured, it cannot be ascertained.
3. A transitional flow is characterized by chaotic eddies and vortices with rapid annihilation and merger of vortices, and their interaction with the flow and each other, which results in distortions within the jet that emanates from the throat, continuous restabilization, and re-disruption of the jet at regular intervals. This results in shifts in the jet breakdown location, which are more pronounced at lower Reynolds numbers close to the *Re*_*crit*_.
4. Questions like *jet breakdown location* have received major attention in the literature. The *Re*_*crit*_, however, should be given equal attention and causes of discrepancies in its identification should be scrutinized to have better understanding of mechanobiological aspects like, for example, hemolysis or endothelial dysfunction.
5. The LBM being a second-order method requires relatively higher mesh density compared with other numerical methods but it can compute flows accurately, and in particular it can predict the onset of transition accurately in a relatively lesser time. On the other hand, the method, if properly implemented keeping HPC in view, scales impeccably on massively parallel computer architectures thereby allowing for simulations of complex geometries at any scale. Whether these facets of the LBM are assets or liabilities would depend on the perspective, the problem in hand, and the research question itself.
6. LBM-based DNS approach would likely become *impractical* for turbulent flows in complex geometries at Reynolds numbers higher than 6500, and complex collision models or turbulence models should be explored in future.

## Electronic supplementary material

Below is the link to the electronic supplementary material.

Vorticity magnitude across a bisecting plane in the FDA nozzle between 9th and 10th second of the simulation of throat Reynolds number 3500, computed from HR LBM simulations. One thousand VTK files were written to disk by the APES framework, which were rendered using Paraview on the SuperMUC-NG (MP4 10.8 MB)

Velocity magnitude across a bisecting plane in the FDA nozzle between 9th and 10th second of the simulation of throat Reynolds number 3500, computed from HR LBM simulations. One thousand VTK files were written to disk by the APES framework, which were rendered using Paraview on the SuperMUC-NG (MP4 8.65 MB)

Vorticity magnitude across a bisecting plane in the FDA nozzle between 9th and 10th second of the simulation of throat Reynolds number 2000, computed from HR LBM simulations. One thousand VTK files were written to disk by the APES framework, which were rendered using Paraview on the SuperMUC-NG (MP4 8.04 MB)

Velocity magnitude across a bisecting plane in the FDA nozzle between 9th and 10th second of the simulation of throat Reynolds number 2000, computed from HR LBM simulations. One thousand VTK files were written to disk by the APES framework, which were rendered using Paraview on the SuperMUC-NG (MP4 6.94 MB)

## Footnotes

1 https://ncihub.org/wiki/FDA_CFD

2 https://apes.osdn.io

3 https://www.paraview.org

4 Note that other works [7, 20] have normalized the *u*_*z*_ with respect to $\bar {u}_{in}$

5 Plots for experiment *#*468 have been omitted as several radial profiles are not available from the data of this experiment.

6 The shear stress at *z*_3_ seems to *go out* of the nozzle. This is a visual artifact as the shear rises dramatically at the walls.

## References

[1] Bergersen AW, Mortensen M, Valen-Sendstad K (2019). The FDA nozzle benchmark: in theory there is no difference between theory and practice, but in practice there is. Int J Numer Methods Biomed Eng.

[2] Bernsdorf J, Harrison SE, Smith SM, Lawford PV, Hose DR (2008). Applying the lattice Boltzmann technique to biofluids: a novel approach to simulate blood coagulation. Comput Math Appl.

[3] Bouzidi M, Firdaouss M, Lallemand P (2001). Momentum transfer of a Boltzmann-lattice fluid with boundaries. Phys Fluids.

[4] Chabannes V, Prud’Homme C, Szopos M, Tarabay R (2017) High order finite element simulations for fluid dynamics validated by experimental data from the FDAbenchmark nozzle model. arXiv:1701.02179

[5] Dellar PJ (2014). Lattice Boltzmann algorithms without cubic defects in Galilean invariance on standard lattices. J Comput Phys.

[6] Delorme YT, Anupindi K, Frankel SH (2013). Large eddy simulation of FDA’s idealized medical device. Cardiov Eng Technol.

[7] Fehn N, Wall WA, Kronbichler M (2019). Modern discontinuous Galerkin methods for the simulation of transitional and turbulent flows in biomedical engineering: a comprehensive LES study of the FDA benchmark nozzle model. Int J Numer Methods Biomed Eng.

[8] Hariharan P, Giarra M, Reddy V, Day SW, Manning KB, Deutsch S, Stewart SF, Myers MR, Berman MR, Burgreen GW (2011). Multilaboratory particle image velocimetry analysis of the FDA benchmark nozzle model to support validation of computational fluid dynamics simulations. J Biomech Eng.

[9] Harlacher D, Hasert M, Klimach H, Zimny S, Roller S (2012) Tree based voxelization of STL data. In: High performance computing on vector systems 2011, pp 81–92

[10] Hasert M, Masilamani K, Zimny S, Klimach H, Qi J, Bernsdorf J, Roller S (2014). Complex fluid simulations with the parallel tree-based lattice Boltzmann solver Musubi. J Comput Sci.

[11] Helgeland A, Mardal K-A, Haughton V, Anders Pettersson Reif B (2014) Numerical simulations of the pulsating flow of cerebrospinal fluid flow in the cervical spinal canal of a Chiari patient. Journal of Biomechanics 10.1016/j.jbiomech.2013.12.023 24529910

[12] Jain K (2020). Transition to turbulence in an oscillatory flow through stenosis. Biomechanics and Modeling in Mechanobiology.

[13] Jain K, Ringstad G, Eide P-K, Mardal K-A (2017). Direct numerical simulation of transitional hydrodynamics of the cerebrospinal fluid in Chiari I malformation: the role of cranio-vertebral junction. Int J Numer Methods Biomed Eng.

[14] Jain K, Roller S, Mardal K-A (2016). Transitional flow in intracranial aneurysms–a space and time refinement study below the Kolmogorov scales using lattice Boltzmann method. Comput Fluids.

[15] Janiga G (2014). Large Eddy simulation of the FDA benchmark nozzle for a Reynolds number of 6500. Comput Biol Med.

[16] Junk M (2011). & Yang, Z. Asymptotic analysis of lattice Boltzmann outflow treatments Communications in Computational Physics.

[17] Klimach H, Jain K, Roller S (2014) End-to-end parallel simulations with apes. In: Parallel computing: accelerating computational science and engineering (CSE), vol 25, pp 703–711

[18] Marié S, Ricot D, Sagaut P (2009). Comparison between lattice Boltzmann method and Navier–Stokes high order schemes for computational aeroacoustics. J Comput Phys.

[19] Nicoud F, Chnafa C, Siguenza J, Zmijanovic V, Mendez S (2018) Large-eddy simulation of turbulence in cardiovascular flows, pp 147–167

[20] Passerini T, Quaini A, Villa U, Veneziani A, Canic S (2013). Validation of an open source framework for the simulation of blood flow in rigid and deformable vessels. Int J Numer Methods Biomed Eng.

[21] Peng C, Geneva N, Guo Z, Wang L-P (2018). Direct numerical simulation of turbulent pipe flow using the lattice Boltzmann method. J Comput Phys.

[22] Pope SB (2000) Turbulent flows. Cambridge University Press

[23] Qi J, Jain K, Klimach H, Roller S (2016) Performance evaluation of the LBM solver Musubi on various HPC architectures. In: Advances in parallel computing: on the road to exascale, volume 27 of advances in parallel computing. IOS Press, pp 807–816

[24] Roller S, Bernsdorf J, Klimach H, Hasert M, Harlacher D, Cakircali M, Zimny S, Masilamani K, Didinger L, Zudrop J (2012) An adaptable simulation framework based on a linearized octree. In: High performance computing on vector systems 2011, pp 93–105

[25] Saqr KM, Mansour O, Tupin S, Hassan T, Ohta M (2019). Evidence for non-newtonian behavior of intracranial blood flow from doppler ultrasonography measurements. Med Biol Eng Comput.

[26] Stewart SF, Paterson EG, Burgreen GW, Hariharan P, Giarra M, Reddy V, Day SW, Manning KB, Deutsch S, Berman MR (2012). Assessment of CFD performance in simulations of an idealized medical device: results of FDA’s first computational interlaboratory study. Cardiovasc Eng Technol.

[27] Succi S (2008). Lattice Boltzmann across scales: from turbulence to DNA translocation. Europ Phys J B.

[28] Succi S, Benzi R, Higuera F (1991). The lattice Boltzmann equation: a new tool for computational fluid-dynamics. Physica D: Nonlinear Phenomena.

[29] Sun C, Munn LL (2008). Lattice-Boltzmann, simulation of blood flow in digitized vessel networks. Comput Math Appl.

[30] Tupin S, Saqr KM, Rashad S, Niizuma K, Ohta M, Tominaga T (2020) Non-Kolmogorov turbulence and inverse energy cascade in intracranial aneurysm: near-wall scales suggest mechanobiological relevance. arXiv:2001.08234

[31] Ventikos Y (2014). Resolving the issue of resolution. Am J Neuroradiol.

[32] White AT, Chong CK (2011). Rotational invariance in the three-dimensional lattice Boltzmann method is dependent on the choice of lattice. J Comput Phys.

[33] Zhang J, Johnson PC, Popel AS (2008). Red blood cell aggregation and dissociation in shear flows simulated by lattice Boltzmann method. J Biomech.

[34] Zmijanovic V, Mendez S, Moureau V, Nicoud F (2017). About the numerical robustness of biomedical benchmark cases: interlaboratory FDA’s idealized medical device. Int J Numer Methods Biomed Eng.

